# Association of circulating inflammatory proteins with type 2 diabetes mellitus and its complications: a bidirectional Mendelian randomization study

**DOI:** 10.3389/fendo.2024.1358311

**Published:** 2024-03-28

**Authors:** Ying-Chao Liang, Ming-Jie Jia, Ling Li, De-Liang Liu, Shu-Fang Chu, Hui-Lin Li

**Affiliations:** ^1^ The Fourth Clinical Medical College of Guangzhou University of Chinese Medicine, Shenzhen, Guangdong, China; ^2^ Department of Endocrinology, Shenzhen Traditional Chinese Medicine Hospital, Shenzhen, Guangdong, China

**Keywords:** Mendelian randomization, bidirectional, type 2 diabetes mellitus, circulating inflammatory proteins, meta-analysis

## Abstract

**Background:**

Increasing evidence indicates that immune response underlies the pathology of type 2 diabetes (T2D). Nevertheless, the specific inflammatory regulators involved in this pathogenesis remain unclear.

**Methods:**

We systematically explored circulating inflammatory proteins that are causally associated with T2D via a bidirectional Mendelian randomization (MR) study and further investigated them in prevalent complications of T2D. Genetic instruments for 91 circulating inflammatory proteins were derived from a genome-wide association study (GWAS) that enrolled 14,824 predominantly European participants. Regarding the summary-level GWASs of type 2 diabetes, we adopted the largest meta-analysis of European population (74,124 cases vs. 824,006 controls) and a prospective nested case-cohort study in Europe (9,978 cases vs. 12,348 controls). Summary statistics for five complications of T2D were acquired from the FinnGen R9 repository. The inverse variance-weighted method was applied as the primary method for causal inference. MR-Egger, weighted median and maximum likelihood methods were employed as supplementary analyses. Results from the two T2D studies were combined in a meta-analysis. Sensitivity analyses and phenotype-wide association studies (PheWAS) were performed to detect heterogeneity and potential horizontal pleiotropy in the study.

**Results:**

Genetic evidence indicated that elevated levels of TGF-α (OR = 1.16, 95% CI = 1.15-1.17) and CX3CL1 (OR = 1.30, 95% CI = 1.04-1.63) promoted the occurrence of T2D, and increased concentrations of FGF-21 (OR = 0.87, 95% CI = 0.81-0.93) and hGDNF (OR = 0.96, 95% CI = 0.95-0.98) mitigated the risk of developing T2D, while type 2 diabetes did not exert a significant influence on said proteins. Elevated levels of TGF-α were associated with an increased risk of ketoacidosis, neurological complications, and ocular complications in patients with T2D, and increased concentrations of FGF-21 were potentially correlated with a diminished risk of T2D with neurological complications. Higher levels of hGDNF were associated with an increased risk of T2D with peripheral vascular complications, while CX3CL1 did not demonstrate a significant association with T2D complications. Sensitivity analyses and PheWAS further ensure the robustness of our findings.

**Conclusion:**

This study determined four circulating inflammatory proteins that affected the occurrence of T2D, providing opportunities for the early prevention and innovative therapy of type 2 diabetes and its complications.

## 1 Introduction

Type 2 diabetes mellitus (T2D) is a chronic metabolic ailment delineated by the dysregulation of systemic glucose homeostasis, and immunometabolic abnormalities assume a pivotal role in the onset and progression of T2D (1, 2). With the evolving lifestyles of the current generation, diabetes has emerged as a significant public health menace. By 2021, 537 million individuals worldwide will endure the affliction of diabetes, with type 2 diabetes comprising over 90% of these instances (3). Persistent hyperglycemia in the bloodstream may engender multi-systemic complications, presenting a notable risk to the patient’s welfare and longevity. A prospective study encompassing 38 nations has revealed that microvascular complications among individuals with T2D affect approximately 18.8% of cases, while macrovascular complications affect roughly 12.7% (4). Global healthcare expenditures for people with diabetes are estimated to be $966 billion in 2021 and are anticipated to exceed $1,054 billion by 2045 (3, 5). Type 2 diabetes and its complications have become a leading cause of disability and mortality worldwide, imposing a substantial financial burden on society in terms of clinical management and treatment. Therefore, it is imperative to seek out potential risk factors for the disease and promptly intervene for prevention and treatment.

Immune responses and inflammatory regulators are strongly associated with pancreatic β-cell dysfunction and insulin resistance in T2D patients. Pro-inflammatory cytokines such as interleukin 1β (IL-1β) can inhibit insulin secretion and pancreatic β-cell proliferation by increasing the transcription and secretion of chemokines (6). Suppressing inflammatory factors in diabetic mice can effectively safeguard islet cells and delay the progression of hyperglycemia (7). The deficiency in macrophage function amplifies systemic insulin sensitivity and diminishes adiposity and hepatic inflammatory response to metabolic stress (8). A meta-analysis of randomized controlled trials has demonstrated that effective anti-inflammatory therapy can substantially decrease the concentrations fasting plasma glucose (FPG), glycosylated hemoglobin (HbA1c), and C-reactive protein (CRP) among patients with T2D, and that individuals with new-onset type 2 diabetes can gain more benefits from this therapeutic approach (9). The pathogenesis of diabetes mellitus involves dysfunction in multiple tissues, and the inflammatory response at the advancement of metabolic disorders exerts its effects in multiple insulin target tissues including fat, liver, and muscle (10, 11). Consequently, exploring circulating inflammatory factors that correspond with the disease phenotype could offer novel targets for preventing and treating type 2 diabetes at the microscopic and molecular levels. Extensive observational evidence suggests a robust correlation between circulating inflammatory cytokines and T2D (12, 13). However, studies with cross-sectional designs are vulnerable to confounding factors and reverse causality, which can impede accurate causal inference. The emergence of proteomics has introduced a new approach to investigating the underlying mechanisms of complex disorders, and the recent influx of large-scale coupling data on genomics and proteomics has enhanced our comprehension on genetic structure of the circulating proteome (14, 15). Genetic determinants of the abundance about inflammation-related circulating proteins may provide valuable insight into type 2 diabetes and its complications (16).

Mendelian randomization (MR) is extensively applied in the etiological studies of diseases as a compelling strategy for exploring causal relationships between exposures and outcomes. It utilizes genetic variation as an instrumental variable, and its structure is founded on the random assignment of alleles during gamete formation and fertilization. Similar to a randomized controlled trial (RCT), this method efficiently eliminates the confounding bias frequently encountered in traditional observational studies (17). Previous research has predominantly concentrated on a few pro-inflammatory cytokines that have an impact on type 2 diabetes and have been restricted to isolated diabetic complications. Nevertheless, the synthesis of systemic inflammation-associated circulating proteins with type 2 diabetes and its common complications has been relatively underexplored. Accordingly, based on a large-scale genome-wide association study (GWAS) of 91 circulating inflammatory proteins, this study employed a bidirectional Mendelian randomization methodology to explore the causal association between inflammatory circulating proteins and type 2 diabetes, and to further examine them in the prevalent complications of type 2 diabetes. This might provide innovative perspectives on future prevention strategies, early interventions, and clinical management of type 2 diabetes.

## 2 Methods

**Figure 1:**
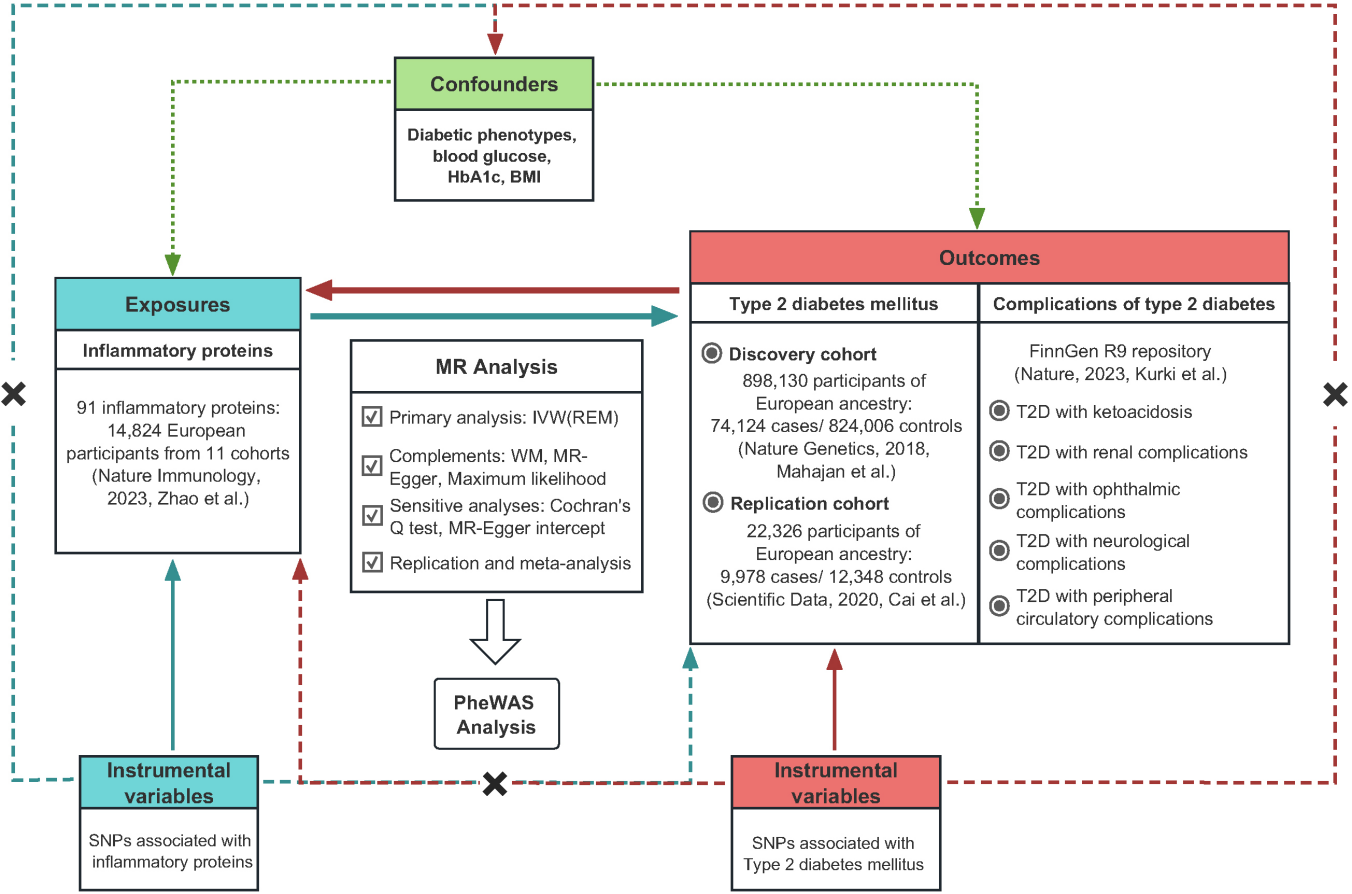
The overall study design regarding the association between circulating inflammatory proteins and diabetic phenotypes.

Mendelian randomization research must fulfill three fundamental assumptions. (I) Instrumental variables (IVs) for genetic variation are tightly related to the exposure. (II) Instrumental variables and confounders are mutually separated. (III) The impact of instrumental variables on the outcome is exclusively mediated through the exposure. The overall structure of this study based on guidelines for conducting Mendelian randomization investigations was displayed in **Figure 1** (17, 18). A total of 91 circulating inflammatory proteins serving as exposures were derived from a recent comprehensive study, and rigorous selection was applied to determine instrumental variables for proxying. We conducted a bidirectional Mendelian randomization study utilizing aggregated statistics from two genome-wide association studies of T2D and further explored in five prevalent complications of T2D. The subject population of this study was limited to participants of European ancestry to reduce potential bias due to demographic stratification. No additional approval from the institutional review board was required since all data utilized in this study originated from studies approved by the respective ethical committees and informed consent was received from all subjects.

### 2.1 Data source for inflammatory proteins

The update GWAS summary statistics for 91 inflammatory proteins were acquired from the study by Zhao et al. (16), which recruited 14,824 predominantly European participants of 11 cohorts. Inflammatory proteins were generated by measuring genome-wide genetic data and plasma proteomics data with the Olink Target Inflammation immunoassay panel. GWAS analysis within each cohort was performed applying an additive genetic association model based on linear regression, and the impact of inflammatory protein was reported as a change in inverse-rank normalized protein level per dosage of the effect allele. The population substructure was adjusted by genetic principal components, and covariates such as age and sex were included in the model to circumvent the effects of possible confounders. Detailed information about quality control and complete data sources are available in the original literature (16).

### 2.2 Data sources for diabetic phenotypes

Summary-level GWASs for type 2 diabetes were obtained from the studies by Mahajan et al. (19) and Cai et al. (20). During the discovery phase, we chose the largest GWAS meta-analysis of European ancestry to date (19). This study involved a total of 74,124 individuals with T2D and 824,006 controls, accessible via DIAbetes Genetics Replication And Meta-analysis consortium (DIAGRAM consortium, https://diagram-consortium.org/). The diagnosis of T2D was in accordance with the clinical criteria of the American Diabetes Association or the World Health Organization, supplemented by healthcare registries, usage of glucose-lowering medications, and validated self-reporting. Patients with suspected type 1 diabetes were excluded on the evidence of GAD antibody and fasting c-peptide levels, premature insulin intervention, and family history. During the replication phase, we adopted summary statistics from a nested case-cohort study of the European Prospective Investigations into Cancer (EPIC) project. The study comprised of 9,978 cases of T2D and 12,348 controls. Detailed study design and data processing could be referred to the original publication (20). FinnGen R9 repository incorporated publicly available GWASs for a variety of diabetic complications, and our study encompassed multiple complications of type 2 diabetes, including nephropathy, ophthalmology, neurology, peripheral circulation, as well as ketoacidosis. Diabetes endpoints were defined implementing World Health Organization recommendations, with the inclusion and exclusion criteria under the International Classification of Diseases (ICD, https://r9.risteys.finngen.fi/) codes, specifically the 10th or 9th revision (21). The diabetic phenotypes and corresponding ICD codes were presented in **Table 1**.

**Table 1:** GWAS sources and data characteristics of type 2 diabetes phenotypes.

Source	Outcome	ICD-10	ICD-9	Phenocode	Ancestry	Cases	Controls	Total samples	Total SNPs
Mahajan et al.	Type 2 diabetes	E11	250.02	–	European	74,124	824,006	898,130	21,602,766
Cai et al.	Type 2 diabetes	E11	250.02	–	European	9,978	12,348	22,326	8,914,240
FinnGen R9 repository	T2D with ketoacidosis	E11.1	2501A	E4_DM2KETO	Finnish	657	308,280	308,937	20,168,576
	T2D with renal complications	E11.2	2503A	E4_DM2REN	Finnish	2,684	308,280	310,964	20,168,475
	T2D with ophthalmic complications	E11.3	2504A	E4_DM2OPTH	Finnish	4,172	308,280	312,452	20,168,538
	T2D with neurological complications	E11.4	2505A	E4_DM2NEU	Finnish	1,894	308,280	310,174	20,168,431
	T2D with peripheral circulatory complications	E11.5	2506A	E4_DM2PERIPH	Finnish	2,179	308,280	310,459	20,168,464

Phenocode corresponds to GWAS identifier in the FinnGen R9 repository.

### 2.3 Selection of instrumental variables

In order to acquire credible instrumental variables, we had devised a series of rigorous procedures of quality control for single nucleotide polymorphisms (SNPs). First, we identified SNPs associated with inflammatory proteins that exhibited genome-wide significance at a threshold of P-value < 5×10-8 to ensure that instrumental variables could proxy for exposure (22). Second, employing a reference panel of European (EUR) populations from the 1000 Genomes project offered by OpenGWAS API (https://gwas-api.mrcieu.ac.uk/) (23), we applied linkage disequilibrium-based clumping with an R^2^<0.001 threshold over a range of 10,000 kb to ensure the isolation of the instrumental variables. Thirdly, we conducted a cross-reference of the selected SNPs with the PhenoScanner database (http://www.phenoscanner.medschl.cam.ac.uk/) to eliminate any SNPs that might conceivably undermine the crucial hypotheses of MR (24). The purpose of this step was to reduce the impact of confounders or the intervention of horizontal pleiotropy. To avoid any potential errors in allele determination and provide accurate causality assessments, we removed palindromic SNPs with uncertain strands and SNPs with non-concordant alleles. Furthermore, we computed the F statistic for each instrumental variable to evaluate their efficacy (R^2^ = 2×EAF×(1-EAF) × beta^2^; F = R^2^ × (N-2)/(1-R^2^)) (25, 26). SNPs with F-statistics < 10 were excluded to mitigate potential bias arising from weak IVs. We eliminated unsuitable IVs according to the exclusion criteria mentioned above and utilized several procedures to guarantee the precision and dependability of our results.

### 2.4 Statistical analysis

#### 2.4.1 Mendelian randomization

Several MR approaches were employed to determine the causal association between circulating inflammatory proteins and type 2 diabetes, as well as its complications. The primary assessment was completed applying the Inverse-Variance Weighted (IVW) approach, yielding general estimates through meta-analysis in combination with Wald ratios for each SNP (27). Compared to the fixed effects model, The IVW method with multiplicative random effects model (REM) could guarantee statistical efficacy even in the presence of weaker random effects (18). In particular, the model with random effects would provide accurate causal estimates even under the interference of horizontal pleiotropy and heterogeneity (18). The weighted median approach yielded precise estimates even when up to 50% of the instrumental variables were unreliable, hence mitigating bias in the evaluation of causal effects in contrast with the IVW method (28). When measurement errors interfered with the exposure effect of SNPs, the maximum likelihood method proved to be more reliable in predicting the parameters of the probability distribution (29). MR-Egger method accepted the presence of pleiotropy and remained valid even when most SNPs exhibited pleiotropic effects, however, the model often lacked statistical performance (30). In comparison to the IVW approach, the other methods exhibited relatively inferior statistical efficacy. Consequently, they were solely applied to corroborate the general direction of the primary method.

#### 2.4.2 Sensitivity analysis

Sensitivity analyses were performed to assess whether independent effects among genetic variables would violate the assumption of Mendelian randomization (31). The IVW method and Egger regression were applied to detect heterogeneity. Cochran’s Q test was implemented to quantify heterogeneity, with instrumental variables with P-value < 0.05 being heterogeneous. The intercept of the Egger regression indicated potential bias in the effect estimates when P-value < 0.05, supporting the horizontal pleiotropy of the instrumental variables. The Steiger filtering was applied to determine the direction of causality between inflammatory proteins and complications of type 2 diabetes, with the assumption that valid genetic variants should explain more variance in the exposure than the outcome (32). The I^2^ and Cochran’s Q tests were employed to examine the heterogeneity of the meta-analysis, with I^2^ greater than 30% considered heterogeneous (16, 33). Effect estimates across different cohorts were combined in meta-analyses adopting either a random-effects model or a fixed-effects model, depending on the presence or absence of heterogeneity.

We implemented the MR program and sensitivity analysis applying the “TwoSampleMR” package in R software (version 4.2.1). The Bonferroni adjustment was adopted to calculate the significance threshold for multiple testing. Inflammatory proteins with a P-value of 5.5e-04 (0.05/91) were defined as significant in the discovery, replication and meta analyses of type 2 diabetes. In complications of T2D, the significance threshold for an adjusted p-value after multiple testing (4 exposures × 5 outcomes = 20 tests) was 0.05/20 = 2.5e-03. Any P-value ranging between the nominal significance (P-value = 0.05) and the Bonferroni correction threshold was deemed indicative of potential causal association. All statistical analyses were two-sided.

### 2.5 Phenome-wide association analysis

To further examine the potential pleiotropy of inflammatory proteins, we conducted a phenotype-wide association analysis on the AstraZeneca PheWAS Portal (https://azphewas.com/) (34, 35). The latest UK Biobank 470k (v5) Public release in AstraZeneca utilized exome sequencing data from 419,391 European participants to investigate the association between protein-coding variants and approximately 10,000 binary and 3,500 continuous phenotypes by phenotype-wide association studies (PheWAS). The significant p-value threshold (1e-8) was established according to the study of Wang et al (35), and corresponded to a false positive rate of 0.1%. The suggestive threshold (1e-6) is adjusted by a single phenotypic collapsing model to preserve conservative control for p < (0.05/18500 genes) (35).

## 3 Results

### 3.1 Determination of instrumental variables

We selected 371 SNPs related to inflammatory proteins in a reliable (P<5×10-8) and independent (R^2^ < 0.001 within 10,000kb) manner using the pooled GWAS of Zhao et al. PhenoScanner excluded 32 SNPs due to their connection with established confounders (diabetes phenotype, glucose, HbA1c, BMI). Seven palindromic SNPs were eliminated due to ambiguity in coordinating allele orientation of the exposure and the outcome. The range of F-statistics for the remaining instrumental variables was 28.98 ~ 2248.71, indicating relatively weak instrumental bias. The characteristics of the determined SNPs were presented in **Supplementary Table S1**.

### 3.2 MR discovery analysis for T2D

**Figure 2:**
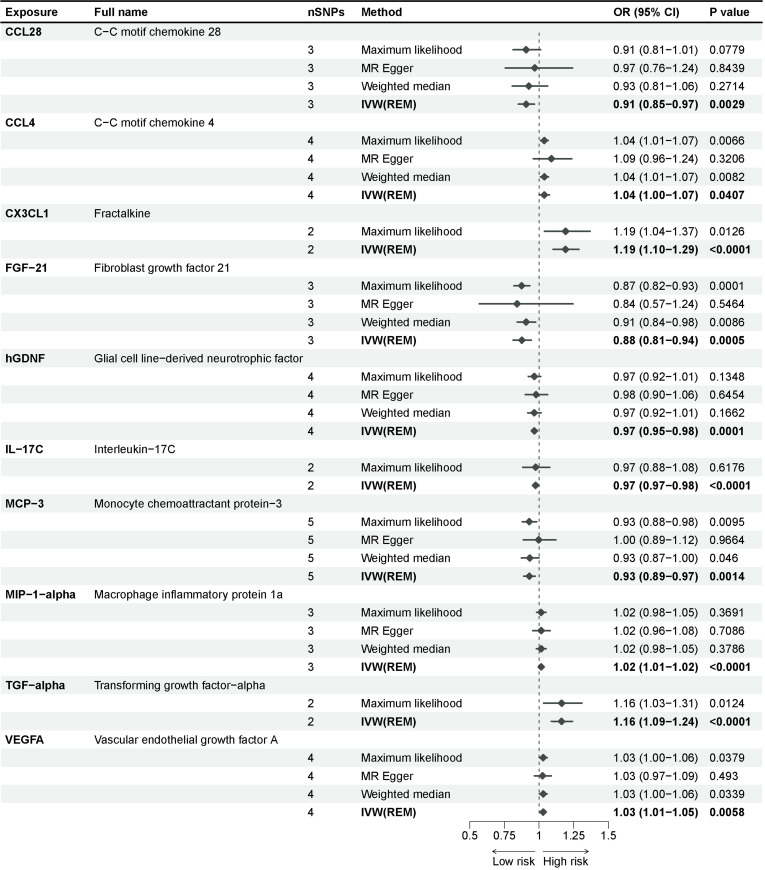
Causal associations between inflammatory proteins and type 2 diabetes in discovery cohorts. The effect of inflammatory proteins on T2D is demonstrated as OR with 95% confidence interval per 1 standard deviation (SD) of concentration change. P-value = 0.0012 (0.05/91) was found significant after multiple-comparison correction.

Adopting the GWAS of Mahajan et al (19), we identified 10 inflammatory proteins underlying a potential causal association with T2D in a multiplicative random-effects model of the IVW method (**Figure 2**). Fractalkine (CX3CL1), fibroblast growth factor 21 (FGF-21), glial cell line-derived neurotrophic factor (hGDNF), interleukin-17C (IL-17C), macrophage inflammatory protein 1a (MIP-1-alpha), and transforming growth factor-alpha (TGF-alpha) exhibited significant effect estimates after Bonferroni adjustment (P-value = 5.5e-04). Only nominally significant P-values were observed for C-C motif chemokine 28 (CCL28), C-C motif chemokine 4 (CCL4), monocyte chemoattractant protein-3 (MCP-3), and vascular endothelial growth factor A (VEGFA). These inflammatory proteins were consistent across other methods in the MR analysis. The maximum likelihood and weighted mean methods yielded identical effect estimates when compared to the IVW method, confirming the robustness of our findings. The odds ratio generated by the MR Egger approach maintained the same trend as the other methods, although it lacked interpretability due to statistical efficacy. The sensitivity analysis of these inflammatory proteins revealed no substantial heterogeneity or horizontal pleiotropy (**Supplementary Table 2**).

### 3.3 MR replication analysis for T2D

**Figure 3:**
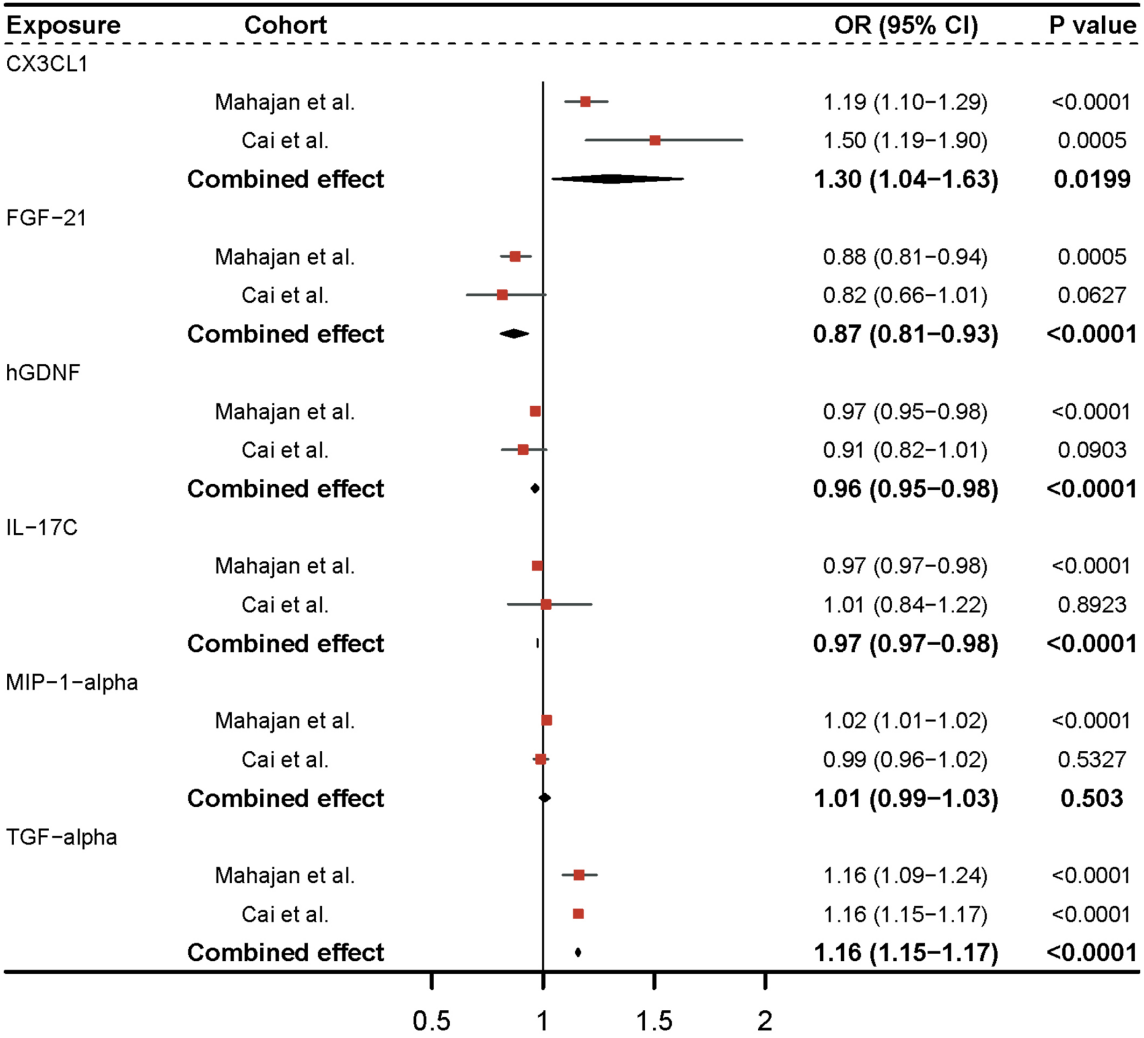
Meta-analysis of causal associations between inflammatory proteins and type 2 diabetes. The results from the IVW method with the multiplicative random-effects model were displayed for all inflammatory proteins.

Utilizing the GWAS of Cai et al (20), we discovered that CX3CL1 and TGF-alpha remained significant after Bonferroni adjustment (P-value = 5.5e-04) in replication analysis for six significant inflammatory proteins (**Figure 3**). Sensitivity analysis revealed no clear evidence of heterogeneity and horizontal pleiotropy (**Supplementary Table 2**). In the meta-analysis of the two cohorts, a random-effects model was employed for CX3CL1 (I^2 =^ 0.72) and MIP-1-alpha (I^2 =^ 0.59) due to the presence of heterogeneity in the two cohorts, and a fixed-effects model was applied for the other inflammatory proteins (**Supplementary Table 3**). Genetic prediction of FGF-21 (OR = 0.87, 95% CI = 0.81-0.93, P-value = 9.77e-05) and hGDNF (OR = 0.96, 95% CI = 0.95-0.98, P-value = 2.77e-05) per a 1-SD increase correspondingly reduced the risk of type 2 diabetes. The combined causal effect of these genes remained significant even after applying the Bonferroni correction (P-value = 5.5e-04). Each SD increase in TGF-alpha (OR = 1.16, 95% CI = 1.15-1.17, P-value = 3.33e-248) and CX3CL1 (OR = 1.30, 95% CI = 1.04-1.63, P-value = 0.0199) was associated with an increased risk of developing type 2 diabetes. The causal estimates for MIP-1-alpha lacked statistical efficacy, and IL-17C was directionally inconsistent in both cohorts. As a result, they were eliminated from subsequent analysis.

### 3.4 Causal effects of T2D on inflammatory proteins

**Figure 4:**
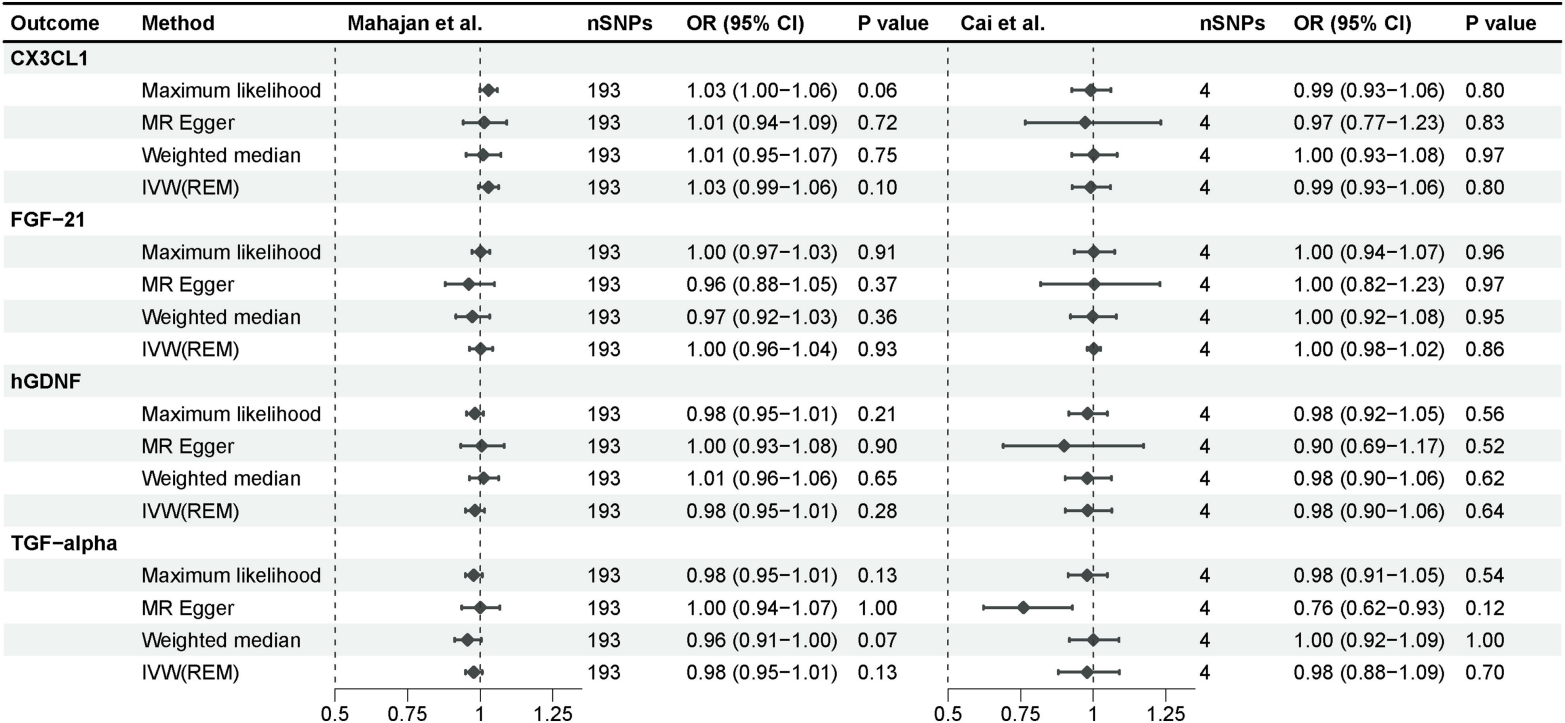
Mendelian randomization analysis of type 2 diabetes on 4 inflammatory proteins. The results derived from the IVW approach using the multiplicative random-effects model were presented.

We investigated the impact of diabetes on identified inflammatory proteins using T2D GWAS as an exposure. According to a similar instrumental variable processing, we selected SNPs that were strongly associated (P-value <5e-8) with type 2 diabetes, and R^2^<0.001 within a 10,000 kb region. After F-statistic exclusion and the coordination of allele SNPs, we acquired 193 SNPs in the study of Mahajan et al. and 4 SNPs in the study of Cai et al. Type 2 diabetes demonstrated no significant effect on CX3CL1, FGF-21, hGDNF, or TGF-alpha across all MR analysis approaches, implying unidirectional causality for our findings (**Figure 4**). The intercept of the Egger regression revealed no horizontal pleiotropy between the two cohorts, and the Cochran’s Q test revealed no heterogeneity in the replication cohort (**Supplementary Table 4**). TGF-alpha exhibited no significant heterogeneity in the discovery cohort, indicating its robustness. CX3CL1, FGF-21, and hGDNF exhibited substantial heterogeneity, which could be attributed to the excessive amount of SNPs in the discovery cohort.

### 3.5 The effect of inflammatory proteins on complications of type 2 diabetes

**Figure 5:**
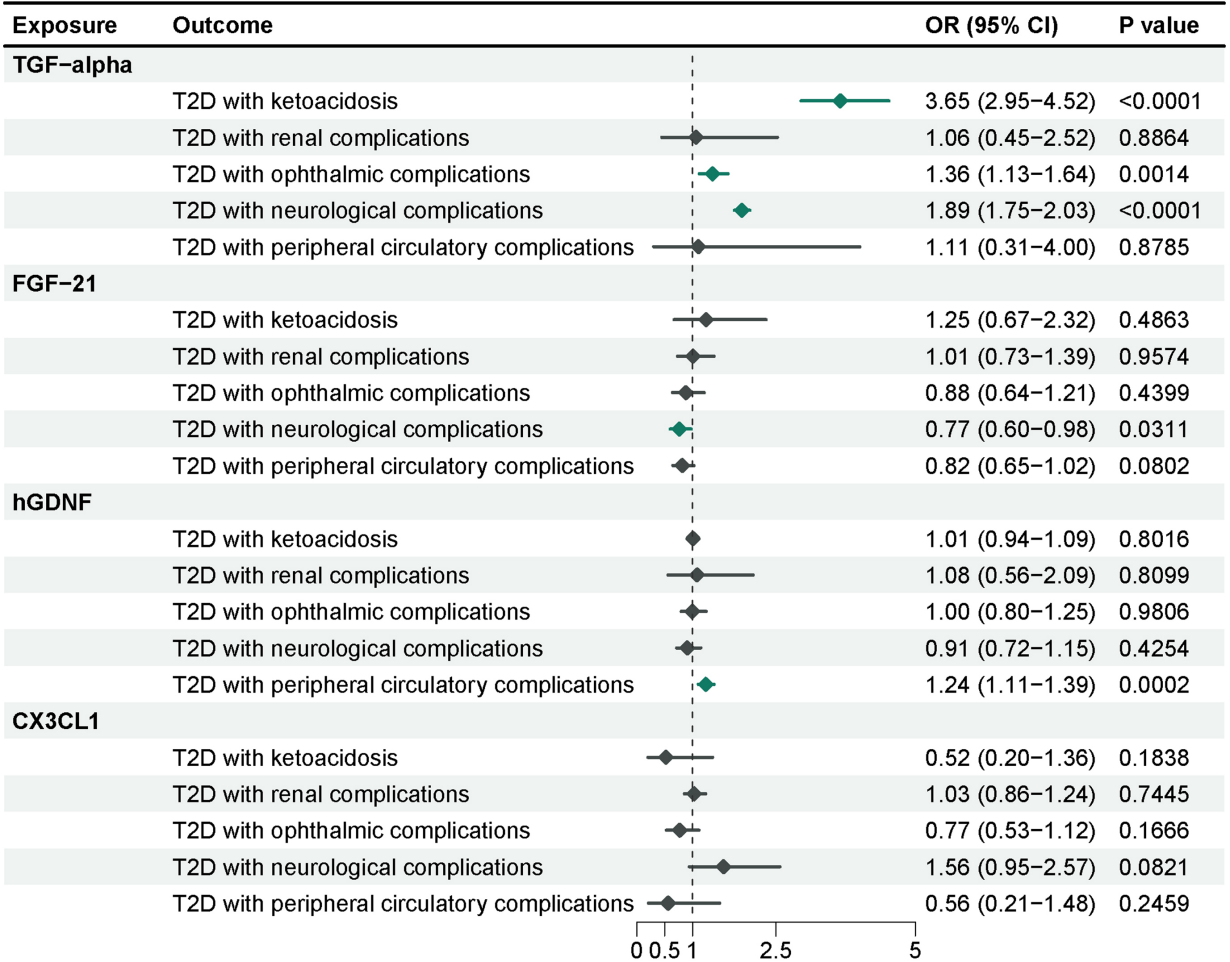
MR analysis of 4 inflammatory proteins on complications of type 2 diabetes. The results from the IVW method with the multiplicative random-effects model were displayed for all inflammatory proteins.

To further investigate the effects of these 4 inflammatory proteins on the advancement of complications of type 2 diabetes, we performed a Mendelian randomization analysis using the pooled GWAS of the FinnGen R9 repository (**Figure 5**) and corrected the IVW results of the multiplicative random-effects model according to Bonferroni method (P-value = 2.5e-03). Genetic evidence indicated that TGF-alpha per a 1 SD increase promoted the occurrence of ketoacidosis (OR = 3.65, 95% CI = 2.95-4.52, P-value = 1.57e-32), neurological complications (OR = 1.89, 95% CI = 1.75-2.03, P-value = 5.13e-65), and ocular complications (OR=1.36, 95% CI =1.13-1.64, P-value= 0.0014) in type 2 diabetes, and there was no significant effect on nephrological complications and peripheral vascular complications. Elevated levels of FGF-21 (OR = 0.77, 95% CI = 0.60-0.98, P-value = 0.0311) reduced the incidence of neurological complications in type 2 diabetes, although this association was only nominally significant. Higher levels of hGDNF (OR = 1.24, 95% CI = 1.11-1.39, P-value = 0.0002) were associated with an increased risk of peripheral vascular complications, and there was no evidence of a significant association between CX3CL1 and complications of type 2 diabetes. Sensitivity analysis indicated no evidence of horizontal pleiotropy. Heterogeneity was observed between hGDNF and T2D with renal complications, as well as TGF-alpha and T2D with peripheral circulatory complications. Steiger test demonstrated a unidirectional causal relationship between these inflammatory proteins and complications of type 2 diabetes in this study.

### 3.6 Phenotype-wide association analysis

To further investigate additional pleiotropy not detected by the MR-Egger intercept test, we conducted a phenotype-wide association analysis at the genetic level using AstraZeneca PheWAS Portal. Only CX3CL1 had a suggestive support for continuous traits in neurological disorders (**Supplementary Figures S1**-**S8**). The absence of significant associations between the 4 inflammatory proteins and any of the binary and continuous phenotypes enhanced the robustness of our findings.

## 4 Discussion

To the best of our understanding, this research represented the inaugural endeavor in systematically determining the causal association between type 2 diabetes and circulating inflammatory proteins. We conducted a bidirectional Mendelian randomization analysis in two independent populations and subsequently examined the impact of noteworthy inflammatory proteins on prevalent complications of type 2 diabetes. Our findings indicated that TGF-α and CX3CL1 exhibited a positive correlation with the genetically predicted susceptibility to T2D, and elevated concentration of FGF-21 and hGDNF could mitigate the risk of developing T2D, while type 2 diabetes did not exert a significant influence on them. Furthermore, TGF-α was revealed to increase the susceptibility to ketoacidosis, neurological complications, and ocular complications in patients with T2D. Increased levels of FGF-21 were potentially correlated with a decreased likelihood of developing neurological complications in T2D patients, and hGDNF elevated the risk of T2D with peripheral vascular complications.

TGF-α belongs to the epithelial growth factor (EGF) family, and it serves to stimulate the aggregation and activation of inflammatory cells, while also inducing the release of inflammatory regulators (36). In high-glucose environments, the induction of TGF-α is stimulated via the hexosamine biosynthesis pathway, leading to insulin resistance in transgenic mice (37). The sustained hyperglycemic state over a brief duration may contribute to TGF-α promoting the onset of ketoacidosis in T2D, especially in individuals with suboptimal glycemic control (38). Notably, among the myriad complications of T2D, the association between TGF-α and retinal disease was particularly intimate. Findings from a prospective case-control study revealed a substantial elevation in TGF-α levels in the vitreous fluid of patients diagnosed with proliferative vitreoretinopathy (39). Factor Xa and thrombin have been substantiated as facilitators of vitreoretinal inflammation and fibrosis processes by regulating TGF-α, ultimately culminating in the manifestation of proliferative diabetic retinopathy (40). The regulation of TGF-α by circRNA and miRNA has been demonstrated to facilitate apoptosis and inflammatory responses in retinal pigment epithelial cells, consequently influencing the advancement of diabetic retinopathy (41, 42). Collectively, these studies indicate that TGF-α plays a crucial role in the pathogenesis of ocular complications of T2D, and targeted intervention against TGF-α emerges as an appealing therapeutic strategy for diabetic retinopathy. TGF-α can alleviate neuroexcitotoxicity and protect neuronal cells from damage by regulating glutamate transporter-1 (GLT-1), presenting a potential novel pharmacological target for the treatment of neurological disorders (43, 44). However, there is a lack of conclusive evidence indicating a connection between TGF-α and the neurological complications of T2D. Therefore, more thorough research in this domain is required.

FGF-21 is an endocrine protein of the fibroblast growth factor (FGF) family, contributing to glycolipid metabolism and anti-inflammatory processes (45). During a randomized controlled trial conducted over an extended period, a decrease in both total and bioactive plasma FGF-21 levels was observed in patients with T2D (46). Liver-derived FGF-21 has the ability to inhibit the glucagon receptor, which in turn promotes β-cell regeneration and improves insulin resistance, ultimately reducing blood glucose levels (47, 48). FGF-21 exhibits potent anti-inflammatory effects in serum and white adipose tissue of mice with T2D. It is capable of suppressing the expression of inflammatory factors induced by insulin resistance, thus improving glucose metabolism in adipocytes (49). Additionally, FGF-21 has been demonstrated to alleviate diabetic neurodegeneration, primarily by diminishing neuroinflammation and oxidative stress, and enhancing the protection of neuronal mitochondria (50). FGF-21 also exhibits a reparative effect on peripheral nerve injury in animal experiments. FGF-21 can inhibit the excessive activation of oxidative stress and autophagy-induced cell death, which is beneficial to myelin re-formation and nerve regeneration after peripheral nerve injury (51). In summary, FGF-21 exhibits remarkable biological efficacy in reducing blood glucose, alleviating inflammation, and ameliorating peripheral nerve injuries, promising to serve as a biomarker and therapeutic target for diabetes management.

hGDNF is a crucial neurotrophic factor, holding a significant position in the intricate domain of medical science (52). A cross-sectional study has discovered that serum hGDNF concentrations in patients with T2D are lower than the controls, and that hGDNF levels are also considerably decreased in individuals with inadequate blood glucose control (53). The overexpression of GDNF in pancreatic glial cells can improve glucose tolerance and significantly promote the survival and proliferation of β-cells (54). In addition, a GDNF family receptor α-like (GFRAL) antagonist can counteract the weight-reducing effect of metformin in obese mice with high-fat diets (55). It is well known that the complications arising in the ocular and renal organs due to type 2 diabetes are inherently microvascular. A prospective case-control study has demonstrated that hGDNF levels were notably elevated in patients with proliferative diabetic retinopathy (56). The expression of GDNF restricts vascular permeability by regulating the function of capillary endothelial tight junctions, thereby influencing the progression of diabetic retinopathy (57). In addition, studies have also demonstrated that GDNF expression can regulate renal microcirculation and inhibit epithelial-to-mesenchymal transition (EndMT) and renal fibrosis (58). These studies indicate that hGDNF holds potential regulatory effects on vascular complications of T2D, but the precise molecular mechanism underlying these effects requires a more profound exploration.

CX3CL1, renowned as Fractalkine, serves as the sole member of the CX3C chemokine family and acts as a specific ligand for the G protein-coupled receptor CX3CR1 (59). A case-control study has revealed that plasma CX3CL1 levels are notably elevated and positively correlated with inflammatory cytokines in patients with T2D compared to the normoglycemic population (60). Another meta-analysis also supports the association between heightened CX3CL1 concentration and the onset of T2D (61), indicating that CX3CL1 may contribute to the pathogenesis of diabetes through inflammation and immune response. An animal experiment has demonstrated that the CX3CL1/CX3CR1 pathway regulates the function of pancreatic β-cell and improves insulin secretion and glucose uptake (62). CX3CL1-CX3CR1, as an inflammatory adipokine chemokine system, can regulate the adhesion of monocytes to adipocytes (63). This signaling pathway modulates adipose tissue inflammation and insulin resistance in obese mice through the recruitment of adipose tissue macrophages and M1/M2 polarization, and therapeutics targeting the CX3CL1-CX3CR1 system may be beneficial for treating type 2 diabetes (64). Furthermore, in adipose tissue, CX3CL1 has been found to influence the progression of T2D by modulating microRNA expression (65). It has been demonstrated that CX3CL1 concentrations manifest a notable elevation in the serum of individuals with impaired glucose tolerance, exhibiting a marked correlation with fasting blood glucose levels (66). Our research indicates that CX3CL1 contributes to the initial onset of type 2 diabetes but exerts an insubstantial effect on the progressive complications of T2D, providing support for the prevention and early intervention of diabetes.

This study possessed several strengths. Firstly, this was the first MR study employing large-scale pooled data systematically to determine the causal relationship between circulating inflammatory proteins and type 2 diabetes as well as its complications. Conventional observational studies were susceptible to reverse causality effects, and we endeavored to prevent interference from reverse causation and minimize residual confounding in this MR study. Secondly, the scrutinized cohorts for the study predominantly comprised individuals of European descent, this was a purposeful selection aimed at mitigating potential biases arising from population stratification. In addition, the coverage of circulating inflammatory proteins within our investigation has been the updated and exhaustive, surpassing any existing compilation. For the outcome, we not only studied type 2 diabetes in two separate cohorts but also further explored multiple common complications. This served to fortify and broaden the research findings. Finally, the serum can be easily obtained in clinical practice without highly invasive procedures, facilitating the clinical promotion and personalized treatment of our study findings. Nevertheless, our study is subject to several limitations. Firstly, the population in this study was limited to participants of European origin, failing to encompass other ethnic populations. That might have neglected genetic and environmental factors unique to other ethnicities, resulting in findings that may not directly apply to populations of different races and regions, thus necessitating additional validation across diverse racial and regional groups. Secondly, we ensured the effective exclusion of confounders through a series of procedures in MR, but there were potential confounders that could affect the accuracy of the study. Consequently, we performed sensitivity analyses and phenotype-wide association analyses to confirm and solidify the reliability of our findings. Thirdly, regarding the circulating inflammatory proteins that did not exhibit statistical significance in this study, it is not possible to rule out a causal relationship between them and type 2 diabetes, potentially due to the constrained SNPs associated with these circulating inflammatory proteins included in the study. In addition, the limited number of SNPs for TGF-alpha and CX3CL1 restricted the application of pleiotropy testing. However, all SNPs associated with these circulating inflammatory proteins served as robust instruments with F-statistics exceeding 10. We also conducted comprehensive phenotype-wide association studies to detect potential horizontal pleiotropy, aiming to reinforce the robustness of our findings. Finally, MR simulated the lifetime low-dose exposure of alleles and assumed a linear relationship between exposure and outcome. However, this method could not fully summarize the clinical trials in the real world. Therefore, MR results should be interpreted as effects during the course of life and might not fully predict the impact of circulating inflammatory proteins on diseases.

## 5 Conclusion

In conclusion, our study revealed pivotal contributions of TGF-alpha, FGF-21, hGDNF, and CX3CL1 in the advancement of type 2 diabetes and its complications. These findings offered favorable implications for the treatment and prevention of diabetes, laying the groundwork for novel clinical approaches and management strategies. However, additional experimental and clinical investigations are necessary to elucidate the functions and molecular mechanisms of these circulating inflammatory proteins in future studies.

## Data availability statement

The survey data of this study are publicly available in the respective genome-wide association studies, and the names of the repository/repositories and accession number(s) can be found in the article/**Supplementary Material**.

## Ethics statement

Ethical approval was not required for the study involving humans in accordance with the local legislation and institutional requirements. Written informed consent to participate in this study was not required from the participants or the participants’ legal guardians/next of kin in accordance with the national legislation and the institutional requirements. All data in this study were generated from studies approved by the respective ethics committees, and informed consent was granted to all subjects. Therefore, no additional approval from the institutional review board was required.

## Author contributions

Y-CL: Conceptualization, Data curation, Formal analysis, Investigation, Methodology, Software, Visualization, Writing – original draft. M-JJ: Data curation, Formal analysis, Investigation, Project administration, Validation, Visualization, Writing – original draft. LL: Conceptualization, Data curation, Methodology, Project administration, Resources, Writing – original draft. D-LL: Funding acquisition, Project administration, Resources, Supervision, Validation, Writing – review & editing. S-FC: Methodology, Project administration, Supervision, Writing – review & editing. H-LL: Funding acquisition, Project administration, Resources, Validation, Writing – review & editing.
