# Supplementary material for: Association of circulating inflammatory proteins with type 2 diabetes mellitus and its complications: a bidirectional Mendelian randomization study
Source: Front Endocrinol (Lausanne). 2024 Mar 28;15:1358311. doi: 10.3389/fendo.2024.1358311 (PMC11007105; doi:10.3389/fendo.2024.1358311)
Supplement: Supplementary file 1 [file DataSheet_1.docx]

**Supplementary figure S1-S8. PheWAS results for the inflammatory proteins. The bottom dashed line represented the suggestive line and the top dashed line was the significant line. Traits that exceeded the significant line were considered to be significantly associated with the gene regulating inflammatory proteins.**

**Figure S1. Binary traits associated with TGFA in PheWAS**

**
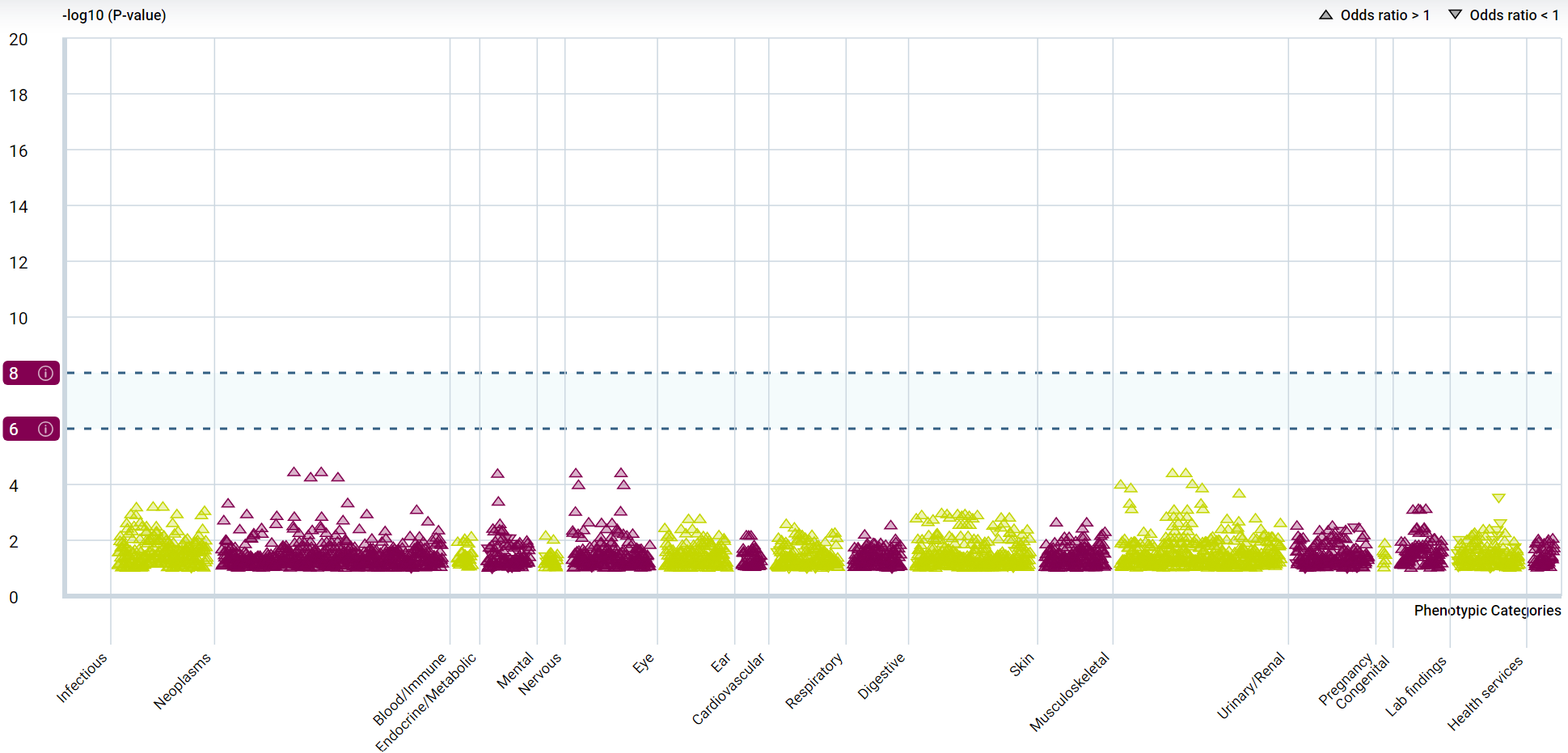
**

**Figure S2. Continuous traits associated with TGFA in PheWAS**

**
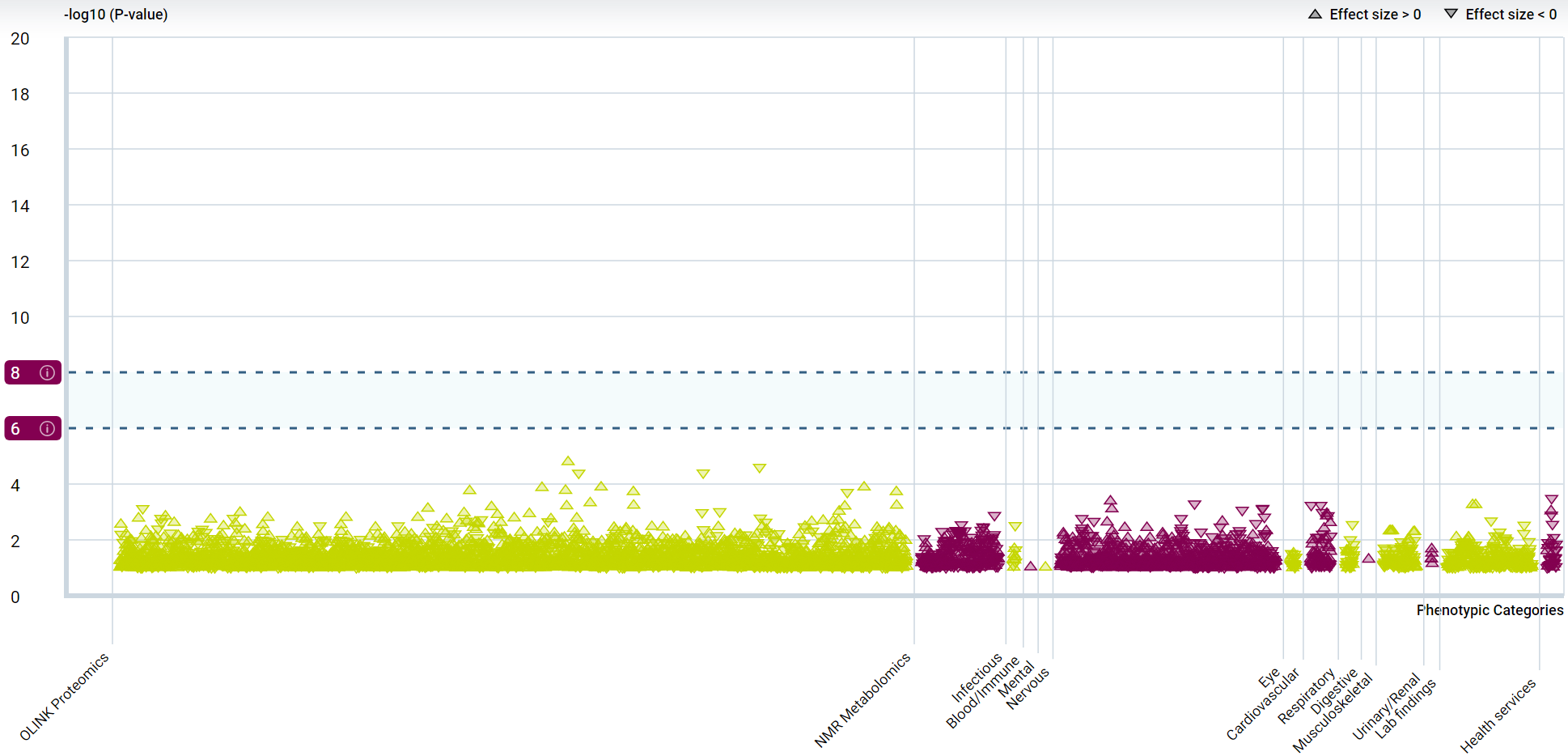
**

**Figure S3. Binary traits associated with CX3CL1 in PheWAS**

**
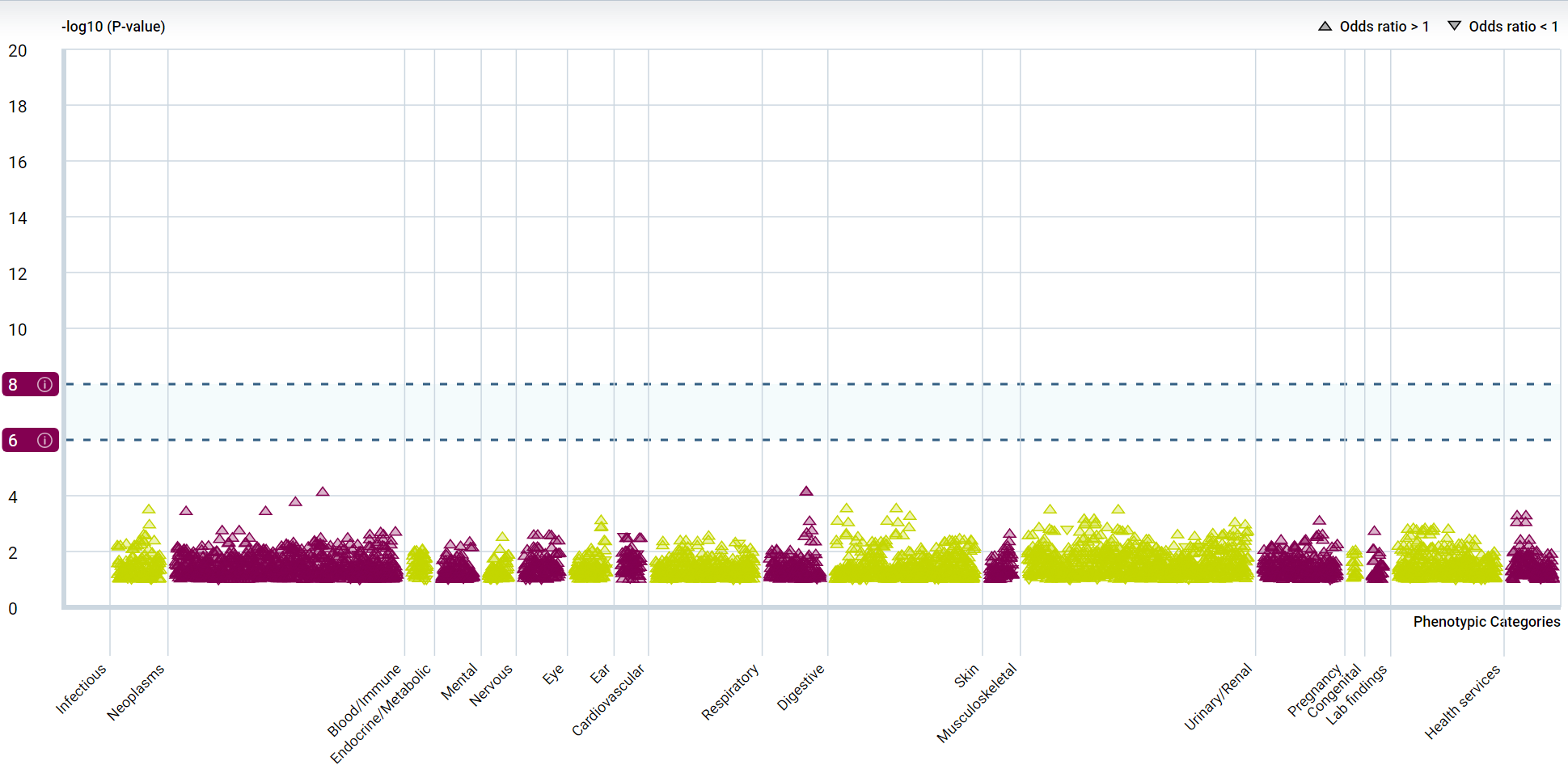
**

**Figure S4. Continuous traits associated with CX3CL1 in PheWAS**

**
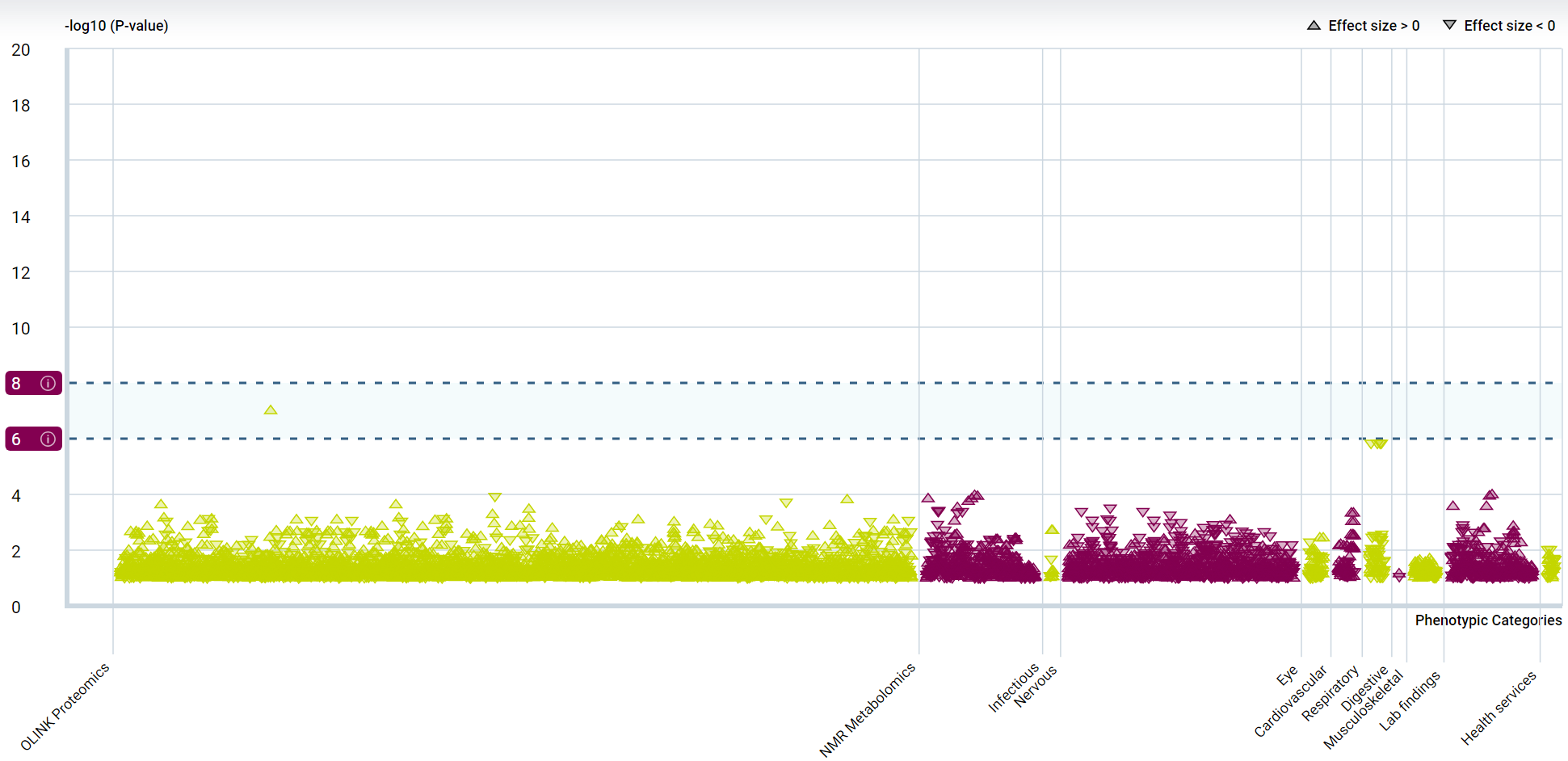
**

**Figure S5. Binary traits associated with FGF21 in PheWAS**

**
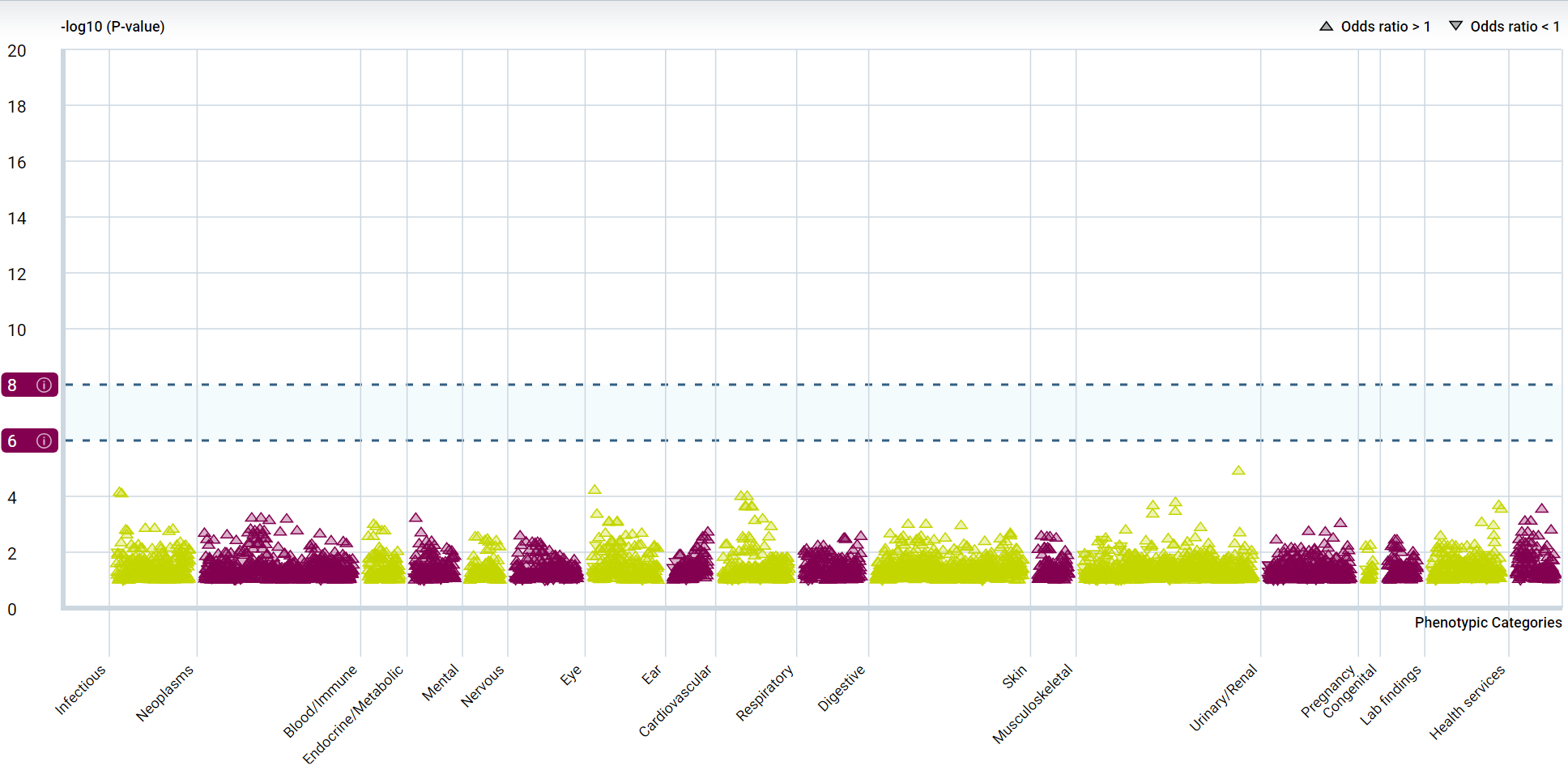
**

**Figure S6. Continuous traits associated with FGF21 in PheWAS**

**
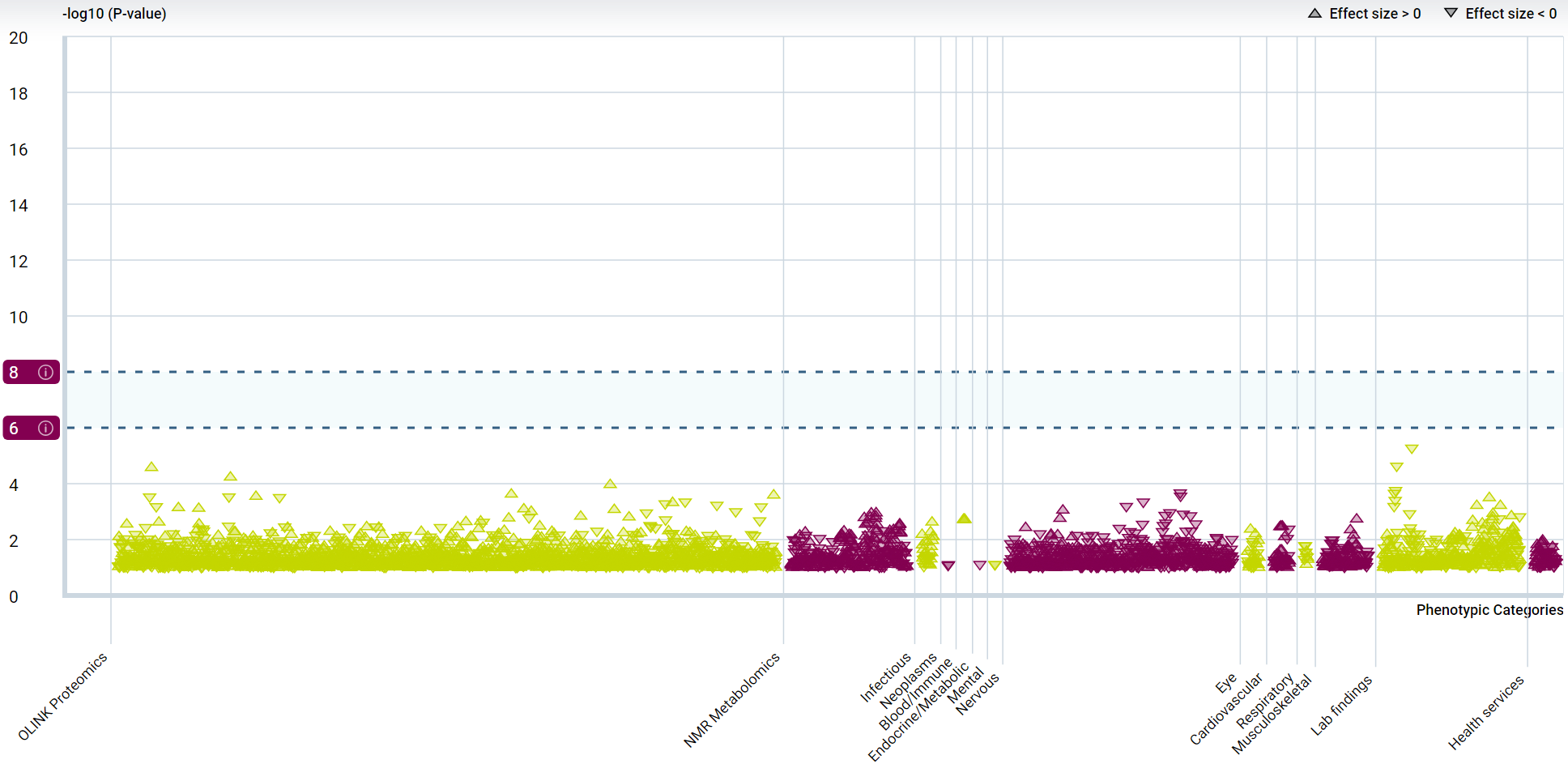
**

**Figure S7. Binary traits associated with GDNF in PheWAS**

**
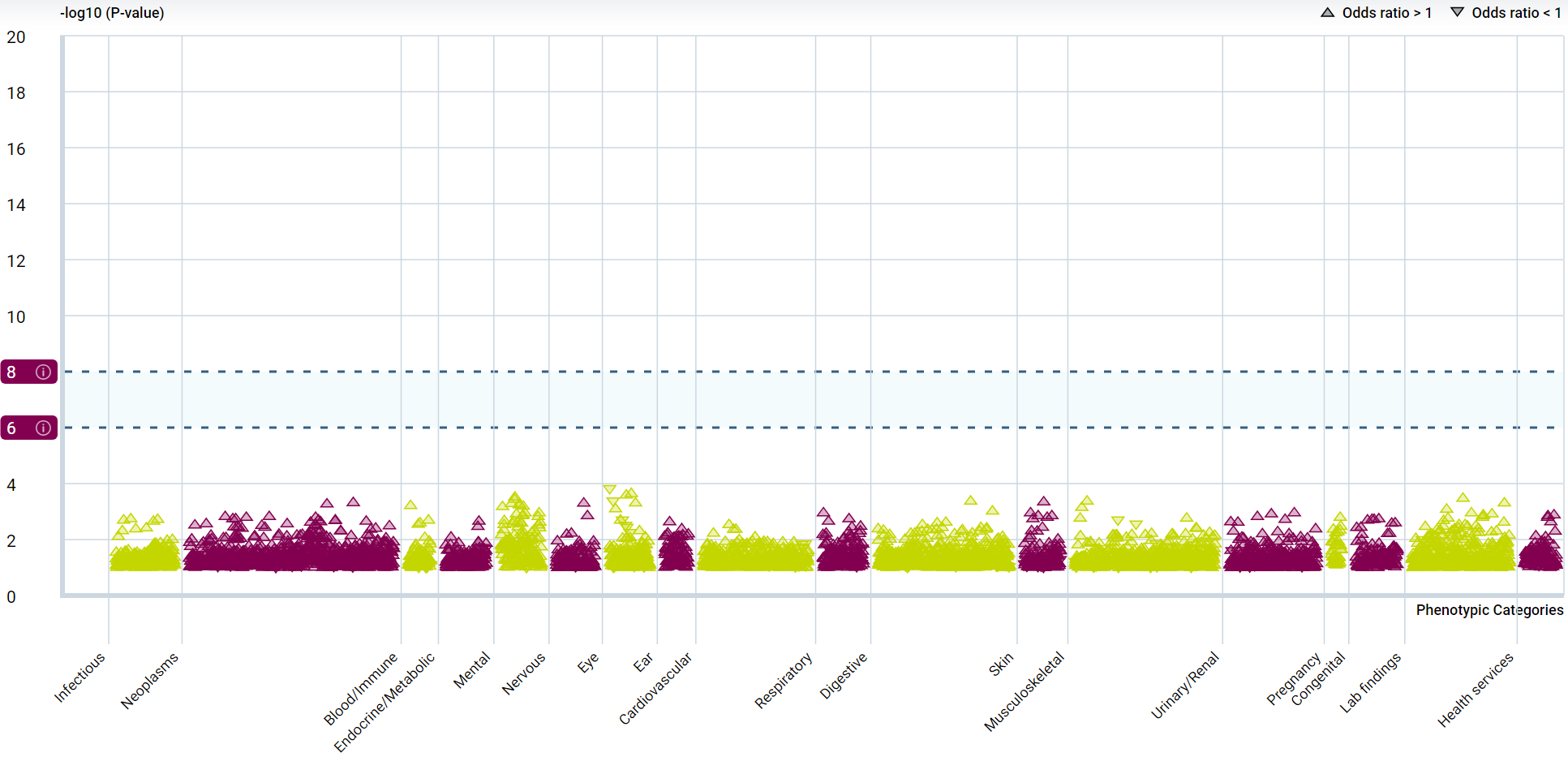
**

**Figure S8. Continuous traits associated with GDNF in PheWAS**

**
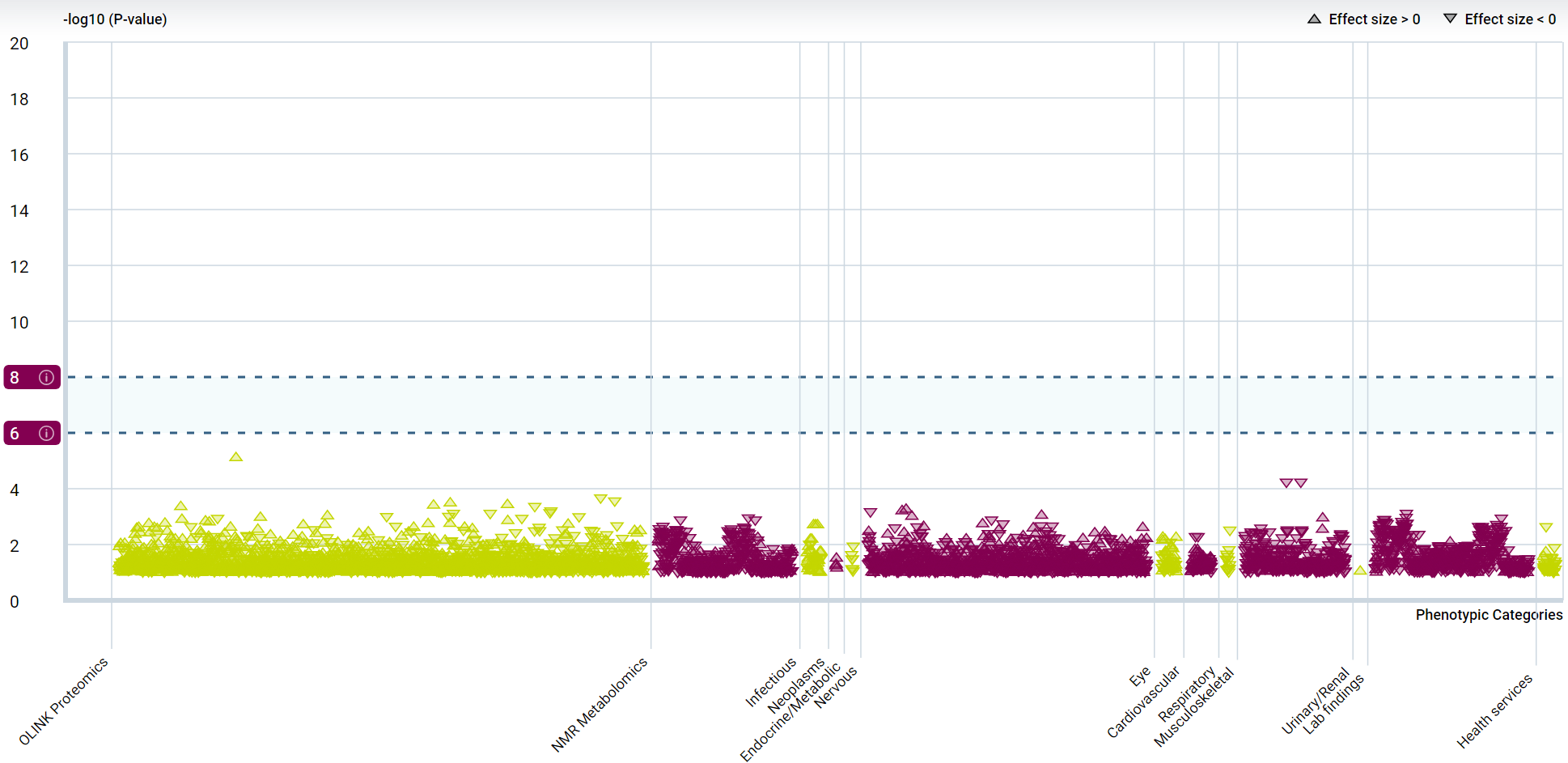
**
